# An ecological dynamics perspective on designing urban nature environments for wellbeing and health-enhancing physical activity

**DOI:** 10.3389/fpubh.2022.877208

**Published:** 2022-07-27

**Authors:** Henrique Brito, Eric Brymer, Duarte Araújo

**Affiliations:** ^1^CIPER, Faculdade de Motricidade Humana, University of Lisbon, Lisbon, Portugal; ^2^Faculty of Health, Southern Cross University, Lismore, NSW, Australia

**Keywords:** ecological dynamics, affordances, wellbeing, physical activity, urban nature environments, urban park design

## Abstract

The latest World Health Organization report on green and blue space and mental health (2021) calls for greater, and better, urban nature environments, i. e., “wilder” urban parks, tree-laden sidewalks, and overall presence of nature in the urban environment. Evidence shows that living close to and interacting with nature promotes benefits to numerous health and well-being indicators. The present article narratively reviews what are the aspects of urban nature environments that enhance health and wellbeing markers, which aspects are preferred among users and visitors of urban nature environments, and how can the benefits for health and wellbeing be understood from a theoretical perspective. Finally, guided by the ecological dynamics framework, suggestions are put forward on how designers and planners of urban nature environments can consider affordances to promote physical activity behavior, health and wellbeing; and how exercise and health researchers and professionals may channel the interaction of individuals with the nature environment in their interventions and programs.

## Introduction

Cities have become massive demographic centers, accelerating urban development. However, the urban landscape has also become a substantial source of pollution, including from traffic and industry (1), which damages air quality, creates noise, pollutes the soil and water, and enables the onset of non-communicable diseases such as cancer, diabetes, cardiovascular and respiratory diseases (2).

The latest report from the World Health Organization (3) about the relationship between green and blue spaces (i.e., environments with a prominence of vegetation and/or water) (3) and mental health concluded that a holistic perspective on urban nature environments (UNE) should be adopted, taking into account health, wellbeing, and ecosystem services contributions (i.e., clean air and water, noise reduction, flood mitigation, ambient temperature reduction, climate stability, biodiversity conservation) (3). This report indicates that there is sufficient scientific evidence to inform policies and initiatives to design the urban environment in ways that improve the health and wellbeing of populations.

Therefore, this article aims to (i) review the literature about the characteristics of UNE which are preferred, and promote health and wellbeing among UNE users; (ii) propose the ecological dynamics framework to understand these benefits; and (iii) offer suggestions about how to design UNE for the promotion of physical activity (PA).

## Urban nature environments – when are they more (or less) healthy?

An insightful distinction of nature and built features was proposed by Wohlwill (4), and later expanded by Heft (5) in which built features are characterized mainly by rectilinear shapes and patterns, smooth surfaces, contrasting colors, abrupt changes in texture and events (e.g., traffic lights suddenly changing), and normative use of environmental features (e.g., how to use a chair). On the other hand, nature features are characterized by curvilinear shapes, rough and irregular surfaces, gradual changes in color and texture, smooth transitions between events (e.g., waves start way back and rockslides take time to end), and no evident normative way to behave or use these features.

Exposure to nature in urban contexts is important for human health and wellbeing (6). Large-scale greenness indexes provided by maps, databases, or global positioning systems, have contributed to finding associations between health indicators and the relative distance and size/quantity of features in UNE (7). For example, Ribeiro et al. (8) found a positive association between distance of schools to UNE and allostatic load (a measure of biological multi-system dysregulation). However, one major challenge in current research is that the concept of ‘exposure to nature' varies in its meaning (9). For example, different green space measures show disparate relationships to PA and obesity (10, 11). To illustrate this, Klompmaker et al. (12) showed that quantifying green space using the Normalized Difference Vegetation Index, *vs*. land-use mapping, resulted in different correlations to engagement in PA in the same sample. Furthermore, these operationalizations often do not consider diversity of flora and fauna inside UNE (13), and, when considered, biodiversity is defined differently in studies because an all-encompassing operationalization is challenging (14).

Other research focuses include the frequency and duration of exposure. Seo et al. (11, 15) conducted a large-scale, 8-year study in Korea, and showed that the people that enjoyed greater green space coverage throughout the 8 years showed reduced risk on the onset of cardiovascular disease. Fisher et al. (16) found a positive association between visits of more than 25 min to UNE and wellbeing. Hunter et al. (17) reported that nature experiences including sitting and walking in UNE for at least 10 min, three times a week, improved wellbeing physiological correlates. At first glance these insights may allow the calculation of an estimation of a health-enhancing dose of nature deriving from the frequency and duration of exposure to UNE (18).

Some recent studies have examined what features of urban parks are preferred by visitors. Liu et al. (19) used photos of urban parks in Shenyang, China, categorized according to the amount of open space, abundance of nature (i.e,. shrubs and trees), and amount of smooth artificial sidewalk. They reported that visitors considered partially open spaces with an abundance of shrubs, trees, and water as superior restorative features compared to paved parks with buildings. Kothencz et al. (20) reported that environment planners in Szeged, Hungary, valued the perception of nature (including biodiversity) and recreational capacity, as some of the most important aspects of visual appearance in UNE. A survey across three Portuguese cities (21) found that respondents rated cleanliness and maintenance, the diversity of plant species, and the existence of water bodies as most important for health and wellbeing.

The ways in which the features of UNE are interpreted by researchers, are quite different. To pursue *exactly what* characteristics of UNE enhance visitor health and wellbeing, studies have explored the quality of nature environments according to specific individuals and populations to clarify the contributions of UNE for health and wellbeing (18, 22). Nonetheless, focusing on individual perceptions, as the majority of existing research has done, such as surveying visitor opinion about the quality of UNE, does not capture other important health-enhancing environmental aspects. Kothencz et al. (20) noted that survey responders seemed to partly dismiss the role of ecosystem services such as noise suppression, microclimate regulation, and air purification. Despite being slow-changing factors, and not as obvious to visitors of UNE as the visual aspect of the environment, ecosystem services play a central role in improving long-term health and wellbeing (20). Although much research on the health and wellbeing influences of nature-based environments is focused on humans as passive receivers of environmental stimuli such as landscape composition and biodiversity (e.g., plants species), dose-response relationship (e.g., time spent in nature environments), accessibility (e.g., proximity to public parks), qualities (e.g., tree canopy coverage), and features (e.g., size) (23), an emphasis on human agency and the relational link between human and environment, i.e., what people can *do* in environments and how it can contribute to well-being, might be more adequate to reveal psychologically relevant properties (24). Following this notion, environmental preference may be related to the kinds of actions that the environment allows a performer to do. Built environments are typically hard and immovable, while nature environments are more prone to change. Facades, signs, trash bins, and walkways are hard to modify and ought not be used for other ends than what they were built for, while in parks, trees, rocks, sticks, and boulders can be climbed on, stacked upon each other, thrown, and used to build structures without other park users looking judgmentally (5, 25). The awareness of ‘agency' in the environment may be an important factor regarding preference and ultimately health and wellbeing (24). Importantly, more than perceptions, an understanding of the quality of nature environments should also consider the perception-action cycle (25, 26).

## UNE and physical activity

Insufficient physical activity is associated with global ill health. UNE promote uptake and maintenance of PA behavior, and also provide added health benefits beyond those found from PA alone (27, 28). Although more investigation is required (29, 30), recent research shows that both the quality and quantity of the UNE is important (7, 31–33). When compared to exercising in settings without nature features (indoor, built), exercising in UNE has shown greater improvements in mood (34), intensity in PA (35), and better physiological health indicators (36), suggesting that there may be a synergistic relationship of UNE and exercise benefits (33). Despite these empirical results, the exact basis for the superior benefits remain unclear. While UNE more generally provide opportunities for active transport, recreation, and greater health and wellbeing benefits than indoor and built settings, understanding exactly how this process works, and how it can be harnessed to increase PA levels, is vital to support the design of effective UNE.

## An ecological dynamics approach to physical activity and wellbeing in UNE

Although UNE design aims to bring nature to the urban environment, they tend to be different than non-urban natural environments, which are not designed. For example, in most UNE, pathways, benches, and flat surfaces are created to allow easy access, places to sit and low risk of accidents. While natural environments may also provide access and places to sit, these are likely to be undulating tracks (if at all), and irregular terrain with intermittent presence of objects such as logs, rocks, trees, and bushes (5).

The process by which nature features enhance wellbeing is not clear, apart from the benefits of ecosystem services (clean air, climate adaptation, etc.), and opportunities for ambulation and recreation (23). The ecological dynamics perspective, with its focus on the individual-environment relationship is ideally suited to explain how UNE might enhance health and wellbeing (25, 37, 38). From this perspective a key explanation for the health and wellbeing benefits of PA behavior in UNE is based on the variability of affordances (i.e., possibilities for action), inherent to the natural environment (25). Although it may seem that sitting possibilities are less obvious in natural environments, because familiar sitting features are missing (e.g., chairs), actually, possible places to sit are more varied in nature because individuals with different capacities and skills are prone to find a suitable feature that affords sitting on, instead of the standard ‘universal' chair which may not conform to ideal criteria for sitting for most users (i.e., they are constrained to find that option to sit due to normative and cultural prescriptions, typical of built environments) (39). Such variability, inherent to natural environments, indicates that different people may perceive different affordances, and consequently will act distinctively, and according to their own characteristics and skills. Importantly, this unmanicured characteristic of the natural environments diversifies the actions a visitor can perform, often suppressed by the presumed notions of how to behave in a conventional, organized, safe, human-made setting such as most UNE. This diversity of affordances induces variability in body sub-system work, which has been connected to better health, cognitive functioning (40, 41) and motor performance (42, 43). Wellbeing is thus derived from the positive feelings of successfully engaging in action possibilities close to one's own capacities and skills, the right level of challenge (24), that is, that they themselves can change the environment and change their relation to its features (5).

A tenet of ecological dynamics is that people, regardless of age, are perceptive systems guided by what they can perform in the environment (25). This implies that organisms do not lose the ability to explore with time, it is their perception-action skills that change over time (e.g., due to training), which influence their ability to navigate the world. Thus, we advocate for an amount of variability that is suited to everyone, to every perception-action skill level, in order to enable different capacities and skills profiles to enjoy challenging physical activity experiences, including older and/or obese people. A rich landscape of affordances offers a variety of courses of action, which can be selected by individuals according to their skills and capacities, when moving in a given context (44). For example, a 20-year-old park visitor may find a faster way through the park by jumping off a lower fence, but an elderly visitor may not perceive such a path through the park and may ‘venture' through the pathways that match their skills and capacities. It is not the case that there are fewer affordances in built environments, however, there are social, normative and cultural constraints to limit novel behavior within built environments (24). Another example is the case of *traceurs* (i.e., *parkour* practitioners). They are characterized by high level perception-action skills, which offer them many ways of moving, by crossing walls, balconies, and other built structures (44, 45). They manage to find variability in behavior in monotonous, stable urban environments, whereas the average citizen will mostly use walkways specifically built for ambulation.

From an ecological dynamics perspective, the design of UNE needs to include affordances for different populations, without a normative purpose for their use, by incorporating aspects of nature, and attracting individuals to engage in PA, enhancing health and wellbeing (44, 45).

## Recommendations for the design of, and programs in, urban nature environments for health-enhancing physical activity

Research suggests that more varied environments result in more varied activities. ‘The end of sitting' was an alternative office experience without chairs or tables. Instead, it was designed with varied shapes and surfaces (46, 47). While working in this office, visitors and workers exhibited varied behavior, transitioning from spot to spot and adopting different postures which translated into more physical activity and varied behavior (48–50).

UNE ought to be designed to promote health-enhancing behavior. Popular incentives for PA such as outdoor gyms seem to be underused (51), with usage rates below 6% of park visitors (51–53), possibly because they offer restricted possibilities for action and repetitive behavior. Outdoor gyms might go unnoticed because they are seen as ‘just another feature' of the park (51). To promote opportunities for PA, UNE need to provide to individuals a diverse and meaningful range of PA modes (44).

Urban park design tends to emphasize aesthetic properties of UNE, which have shown to increase park visitation (54). However, not enough attention is paid to the functional relationship of the individuals with the physical features of UNE, to allow for PA behavior to occur (44). The aesthetic emphasis is prone to create UNE that afford escapist activities, such as contemplation and nature appreciation. Although they afford improvements to wellbeing, these activities do not promote PA behavior, because the focus is put on comfort, shelter, safety, and refuge (55).

To create affordances for PA behavior in UNE, ideas for park design can be extrapolated from children's playgrounds, which have been the subject of extensive analysis in the literature over the years. An analysis of van Eyck's playgrounds shows that the abstract sculptures, typical from these parks, were used to stimulate children's creativity in order to invite them to engage in exploration of what they could do with the equipment (56). Contrasting with this view, modern playgrounds had standardized distances between jumping blocks, and climbing bars, which might be aesthetically appealing, but do not elicit movement variability and creativity to playing children (57). Indeed, further studies have shown that children often design their own, irregular playgrounds, according to their skills and capacities (58). Thus, increasing variability in park paths may not attract risk seekers only (e.g., *traceurs*), but all park users as long as there is a wider offer of PA opportunities for people with different movement skills and capacities (45).

For the casual park visitor, the affordances of built features must be accessible, yet challenging, and in harmony with the variability of nature affordances (25). So, urban parks can benefit from design features that allow visitors to explore different forms of PA, that actually resemble an exploratory activity such as parkour, in the sense of activating all cognitive and perceptual-motor capacities (59). This could be achieved, for example, by adopting a concept of ‘*all roads lead to Rome*', where several pathways that would end up at the same place, or at an exit of the park, would demand different skills and capacities, from the standard flat road, to the irregular cobbled road, the uphill dirt track, the scattered rock path crossing a water stream, the small knee-high bush fence obstacle, the climbing wall over a bush fence, to the climbing bars over a small chasm. This is analogous to tracks with different levels of difficulty, which are already imbedded in the design of ski resorts. These different courses would ideally encompass all profiles of park users including children, adolescents, adults, older adults, sedentary and obese people, and others. This is the strength of framing our proposal by ecological dynamics. It allows us to design the urban environment by taking into account the way park users perceive the environment and how they decide to act in it, which is a step up from previous frameworks which apparently have not succeeded in improving physical activity levels worldwide.

Exercise and health researchers studying the benefits of UNE may expand these ideas to green care interventions and green social prescribing, which are nature-based therapies or treatment interventions “specifically designed, structured and facilitated for individuals with a defined need” (45, p. 100, citing 46). To potentiate the interaction between the setting and the mindset, these health interventions are based on different ways of experiencing nature that escalate from passive experience of a natural environment (healing gardens, greening the built environment, ‘view from a window'), to being active within a natural green environment (animal-assisted interventions, green exercise interventions, wilderness therapy), and to shaping the natural green environment (care farming, environmental conservation interventions, horticulture-based approaches) (60). In short, the ways of acting in nature may benefit from variability at both sides: variability in how nature is presented, and variability in how to act on nature.

## Conclusion

The WHO's 2030 global action plan for physical activity (61) upholds that the adoption of active lifestyles in harmony with nature is imperative to improve public health. To achieve this goal, it is necessary that UNE are designed to promote PA and be extended to more urban territory. Furthermore, access to nature environments is related to socio-cultural and socio-economic factors, which means that the poorer strata of society usually live in less green neighborhoods, and therefore have fewer opportunities to visit and enjoy its benefits (23). These social issues, which have repercussions on public health, can be tackled with the implementation of a greener, sustainable environment, spread throughout the urban territory, regardless of socioeconomic status (62), since vegetation, located generally throughout the urban environment, not just in parks, can improve health (9, 63), and reduce mortality in city populations (64).

To promote PA, planners of UNE can design semi-open spaces that allow recreation, together with designed pathways to reach areas or exits in the park, and more bushes and trees, i.e., a “wild urban nature” (65), which would invite biodiversity, diverse movement interactions and promote health and wellbeing. Furthermore, standard outdoor gyms may be modified to provide exploration, variability and diversity in movement, adequately framed by exercise and health professionals.

## Data availability statement

The original contributions presented in the study are included in the article/supplementary material, further inquiries can be directed to the corresponding author/s.

## Author contributions

HB, EB, and DA contributed to conception, design of the study, and the literature review. HB wrote the first draft of the manuscript. EB and DA wrote sections of the manuscript. All authors contributed to the article and approved the submitted version.

## Funding

HB and DA were partly funded by the Fundação para a Ciência e a Tecnologia [grant numbers UIDB/00447/2020, SFRH/BD/147966/2019, and UIDB/00477/2020 awarded to CIPER- Centro Interdisciplinar para o Estudo da Performance Humana (unit 447)].

## Conflict of interest

The authors declare that the research was conducted in the absence of any commercial or financial relationships that could be construed as a potential conflict of interest.

## Publisher's note

All claims expressed in this article are solely those of the authors and do not necessarily represent those of their affiliated organizations, or those of the publisher, the editors and the reviewers. Any product that may be evaluated in this article, or claim that may be made by its manufacturer, is not guaranteed or endorsed by the publisher.
